# Effect of Direct-Acting Antiviral Therapy on Glycemic Control in Patients with Chronic Hepatitis C and Type 2 Diabetes: A Systematic Review and Meta-Analysis

**DOI:** 10.3390/v18020239

**Published:** 2026-02-13

**Authors:** Jing-Hong Hu, Ming-Ling Chang, Ming-Shyan Lin, Tung-Jung Huang, Yung-Yu Hsieh

**Affiliations:** 1 Division of Gastroenterology and Hepatology, Yunlin Chang Gung Memorial Hospital, Yunlin 638, Taiwan; 2 Division of Gastroenterology and Hepatology, Linkou Chang Gung Memorial Hospital, and College of Medicine, Chang Gung University, Taoyuan 333, Taiwan; mlchang8210@gmail.com; 3 Division of Cardiology, Chiayi Chang Gung Memorial Hospital, Chiayi 613, Taiwan; mingshyan@gmail.com; 4 Department of Internal Medicine, Yunlin Chang Gung Memorial Hospital, Yunlin 638, Taiwan; donaldhuang@cgmh.org.tw; 5 Department of Medicine, College of Medicine, Chang Gung University, Taoyuan 333, Taiwan; 6 Department of Respiratory Care, Chang Gung University of Science and Technology, Chiayi 613, Taiwan; 7 Division of Gastroenterology, Chiayi Chang Gung Memorial Hospital, Chiayi 613, Taiwan; ivan5510@gmail.com

**Keywords:** hepatitis C, direct-acting antivirals, type 2 diabetes mellitus, glycemic control, HbA1c, meta-analysis

## Abstract

The eradication of hepatitis C virus (HCV) with interferon-free direct-acting antivirals (DAAs) has transformed the management of chronic HCV infection. Chronic HCV infection is associated with an increased risk of type 2 diabetes mellitus (T2DM) and poor glycemic control. However, the magnitude and consistency of improvement in glycated hemoglobin (HbA1c) after DAA-induced sustained virologic response (SVR) in patients with established T2DM remain unclear. We conducted a systematic review and meta-analysis of six cohort studies comprising 2805 patients. Overall, DAA therapy was associated with a significant reduction in HbA1c after SVR, with a pooled random-effect mean difference of −0.45% (95% CI −0.74% to −0.16%; I^2^ = 97.8%). This effect is highly heterogeneous but suggests that HCV may be a modifiable contributor to chronic hyperglycemia. These findings highlight the need for close glucose monitoring and individualized adjustment of antidiabetic therapy after SVR to optimize metabolic outcomes.

## 1. Introduction

Chronic hepatitis C virus (HCV) infection is a major global health burden, affecting an estimated 58 million people worldwide and contributing substantially to cirrhosis, hepatocellular carcinoma, and liver-related mortality [1]. Beyond hepatic injury, HCV is now recognized as a systemic disease with multiple extrahepatic manifestations, including mixed cryoglobulinemia, renal disease, cardiovascular complications, and an increased risk of type 2 diabetes mellitus (T2DM) [2,3]. Epidemiological studies and meta-analyses have demonstrated that individuals with chronic HCV infection have a higher prevalence and incidence of T2DM than non-infected or hepatitis B virus-infected counterparts [2,3].

The bidirectional association between HCV and T2DM appears to be mediated largely through HCV-induced insulin resistance (IR) and steatosis [2,3,4,5]. Experimental and clinical data indicate that HCV can directly interfere with insulin signaling pathways, upregulate pro-inflammatory cytokines, induce oxidative stress, and promote hepatic steatosis, thereby contributing to systemic IR and β-cell dysfunction [4,5]. As a result, patients with chronic HCV infection are at increased risk of developing T2DM and often experience suboptimal glycemic control when diabetes is present [2,3,4,5].

Interferon-free direct-acting antiviral (DAA) therapies have revolutionized HCV management, achieving sustained virologic response (SVR) rates exceeding 95% across a broad spectrum of patients, including those traditionally considered difficult to treat [6]. In addition to preventing progression to cirrhosis and hepatocellular carcinoma, viral eradication with DAAs has the potential to ameliorate extrahepatic complications such as IR, T2DM, and cardiovascular risk [5,6,7]. Several cohort studies have reported improved glycemic control following DAA therapy in patients with chronic HCV infection and established T2DM, but the magnitude, durability, and consistency of these changes have varied across populations and study designs [7,8,9,10,11,12].

While previous reviews have explored the link between HCV and metabolic disorders, our study provides a more focused and rigorous quantitative synthesis by exclusively including studies that (1) enrolled patients with clearly defined, pre-existing T2DM; (2) utilized interferon-free DAA regimens only; and (3) reported explicit pre- and post-treatment HbA1c values, allowing for the precise estimation of the treatment effect. By conducting a comprehensive meta-analysis with extensive subgroup and sensitivity analyses, we aim to clarify the magnitude of glycemic improvement and provide robust evidence to guide clinical practice in this specific patient population [1,2,3,6,7,8,9,10,11,12,13,14].

## 2. Materials and Methods

### 2.1. Search Strategy and Study Selection

This systematic review was conducted in accordance with the Preferred Reporting Items for Systematic Reviews and Meta-Analyses (PRISMA) 2020 statement [13]. The protocol was registered in PROSPERO (CRD420251110088). We systematically searched PubMed, Embase, and the Cochrane Library from database inception to July 2025. The search strategy combined controlled vocabulary and free-text terms related to “hepatitis C virus”, “direct-acting antivirals”, “type 2 diabetes mellitus”, “glycemic control”, and “HbA1c”, and was developed with the assistance of an experienced medical librarian [13]. Reference lists of relevant articles and prior reviews were also screened to identify additional eligible studies [6]. The detailed search strategy is provided in Supplementary Table S1.

Studies were included if they met all of the following criteria: (1) adult patients with chronic HCV infection and clearly defined T2DM; (2) treatment with interferon-free DAA regimens; (3) reporting quantitative HbA1c values before and after DAA therapy, or sufficient data to derive changes in HbA1c; (4) achievement of SVR, typically defined as undetectable HCV RNA at least 12 weeks after completion of DAA therapy (SVR12); and (5) a minimum post-treatment follow-up duration of 12 weeks [7,8,9,10,11,12]. We excluded studies limited to patients with type 1 diabetes or without a clear T2DM definition, studies using interferon-based or mixed interferon-DAA regimens without separable DAA-only data, case reports or small case series (<10 patients), conference abstracts without accessible full texts, and non-original articles such as narrative reviews or editorials [6].

### 2.2. Data Extraction and Quality Assessment

Two reviewers independently screened titles and abstracts, assessed full texts for eligibility, and extracted data using a standardized form (Supplementary Table S3) [7,8,9,10,11,12]. Extracted information included study characteristics (first author, year of publication, country, study design, setting, and sample size), patient demographics (age, sex, and body mass index), diabetes-related variables (duration of T2DM, baseline HbA1c, and reported changes in glucose-lowering therapy during follow-up), HCV-related variables (genotype, baseline viral load, liver fibrosis stage, presence of cirrhosis, and history of hepatic decompensation), DAA regimens (agents, treatment duration, and SVR rates), and outcomes (pre- and post-treatment HbA1c means and standard deviations, follow-up duration, and adverse metabolic events) [7,8,9,10,11,12].

Study quality and risk of bias for observational cohort studies were evaluated using the Newcastle–Ottawa Scale (NOS), which assesses three domains: selection of cohorts, comparability of groups, and ascertainment of outcomes [14]. Each study was assigned up to nine stars, with higher scores indicating better methodological quality. Any discrepancies in study selection, data extraction, or risk-of-bias assessment were resolved through discussion, and if disagreement persisted, a third senior reviewer adjudicated. The detailed risk of bias assessment for each study is presented in Supplementary Table S2.

### 2.3. Statistical Analysis

For continuous outcomes such as glycated hemoglobin (HbA1c), we calculated the mean difference (MD) in HbA1c (post- minus pre-treatment) for each study, with negative values indicating improved glycemic control [7,8,9,10,11,12]. When studies reported only separate pre- and post-treatment means and standard deviations (SDs) but not the SD of the change, we derived the SD of the change using a standard formula and assumed a conservative correlation coefficient of 0.5 between paired measurements, consistent with prior meta-analyses evaluating changes in HbA1c. To assess the robustness of this assumption, we performed sensitivity analyses using alternative correlation coefficients of 0.3 (lower correlation) and 0.7 (higher correlation) to examine the impact on pooled estimates and heterogeneity [15].

We pooled study-specific MDs using a random-effect model (DerSimonian–Laird), given the anticipated clinical and methodological heterogeneity across studies [15,16]. Statistical heterogeneity was assessed using Cochran’s Q test and quantified by the I^2^ statistic, with values >50% indicating substantial heterogeneity [15]. Sensitivity analyses using fixed-effect models were performed to examine the robustness of the findings. Where data permitted, subgroup analyses were conducted according to baseline HbA1c (e.g., poorly controlled vs. moderately controlled T2DM), presence of cirrhosis, and specific DAA regimens [5,7,8,9,10,11,12,13]. All analyses were performed using standard meta-analytic techniques implemented in R 4.3.2 (R Foundation for Statistical Computing) with the “metafor” package [16]. The R code used for the analysis is provided in Supplementary File S1.

## 3. Results

### 3.1. Study Selection and Characteristics

**Figure 1:**
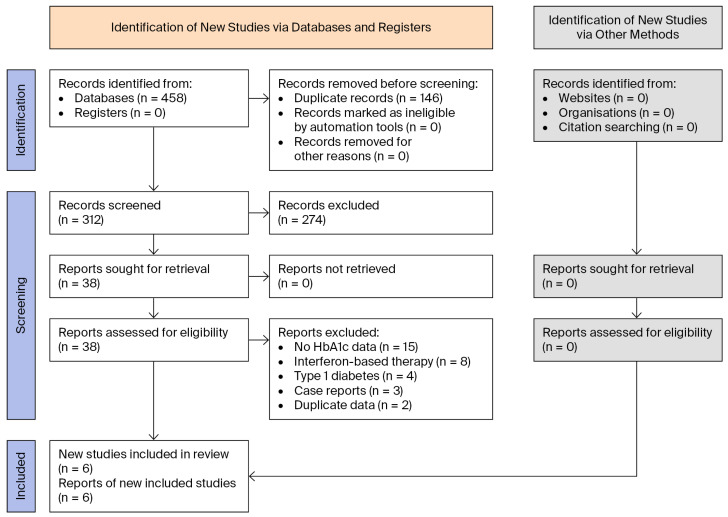
PRISMA 2020 flow diagram. Note: records excluded after full-text review were primarily due to non-eligible populations (e.g., no established T2DM), non-eligible outcomes (no pre-/post-DAA HbA1c or glycemic endpoints), non-DAA regimens/era, insufficient extractable data, duplicate cohorts, or non-original research (reviews/editorials/conference abstracts).

The literature search identified 458 potentially relevant articles. Following removal of duplicates and screening of titles and abstracts, 38 full-text articles were assessed for eligibility, of which six cohort studies met the inclusion criteria and were included in the quantitative synthesis [7,8,9,10,11,12,13]. The study selection process is summarized in the PRISMA 2020 flow diagram (Figure 1) [13].

The six included studies were conducted between 2017 and 2021 in Italy, Japan, the United States, and Egypt [7,8,9,10,11,12]. All were observational cohort studies (four retrospective and two prospective) that enrolled adults with chronic HCV infection and established T2DM who received interferon-free DAA regimens and achieved SVR [7,8,9,10,11,12]. Sample sizes ranged from several dozen to over 1000 participants, yielding a combined total of 2805 patients [7,8,9,10,11,12]. Key study characteristics, including study design, country, sample size, DAA regimens, follow-up duration, and main outcomes, are summarized in Table 1.

Detailed patient demographics and clinical features are presented in Table 2. The pooled cohort had a mean age of 58.4 ± 8.2 years, with 59.0% male participants. The mean baseline HbA1c was 7.8 ± 1.4%, and 44.0% of patients had cirrhosis. The majority of patients were infected with HCV genotype 1 (65.0%), and the most commonly used antidiabetic medication was metformin (75.0%). Baseline HbA1c levels were generally above the recommended targets for T2DM, reflecting suboptimal glycemic control, and the prevalence of advanced liver disease, metabolic syndrome, and other comorbidities varied across studies [5,7,8,9,10,11,12,13].

Overall methodological quality was high, as assessed using the Newcastle–Ottawa Scale (NOS) and detailed in Table 3. Four studies received the maximum score of 9/9 and were rated as high-quality, while two studies scored 7/9 and were rated as moderate-quality, with NOS scores ranging from 7 to 9, indicating low-to-moderate risk of bias [14].

### 3.2. Effect of DAA Treatment on HbA1c

**Figure 2:**
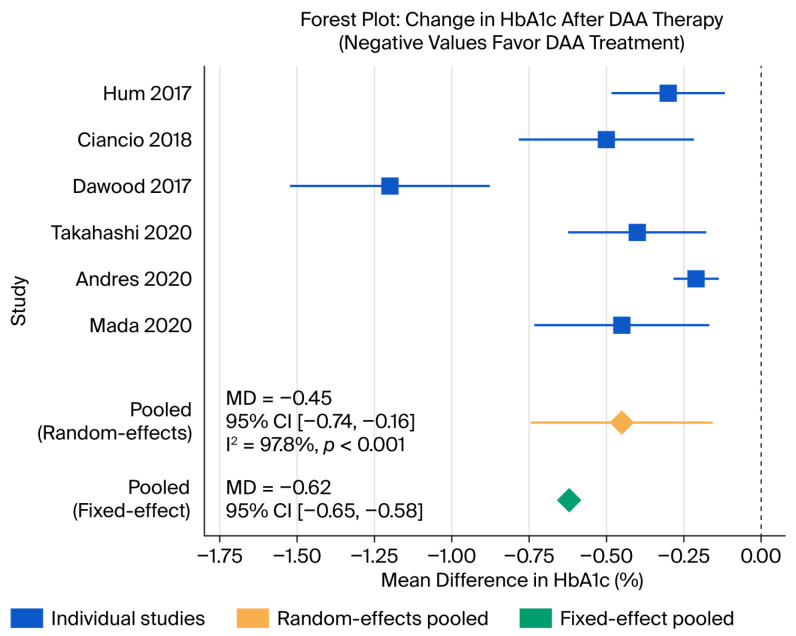
Forest plot of HbA1c change after DAA therapy [7,8,9,10,11,12]. The dashed line represents no difference in HbA1c. The box and whiskers represent MD and the upper/lower bounds of 95% CI, respectively, of each study. CI: confidence interval; DAA: direct anti-viral agent; MD: mean difference.

All six studies reported changes in HbA1c before and after DAA treatment in patients with chronic HCV infection and T2DM [7,8,9,10,11,12]. As shown in the main forest plot (Figure 2), the random-effect meta-analysis demonstrated that DAA therapy was associated with a statistically significant reduction in HbA1c after SVR, with a pooled mean difference (MD) of −0.45% (95% CI −0.74% to −0.16%). However, heterogeneity across studies was substantial (Q = 223.5, *p* < 0.001; I^2^ = 97.8%), reflecting variability in baseline HbA1c, diabetes duration, cirrhosis status, DAA regimens, and follow-up durations. The study by Dawood et al. [10] appeared to be a significant outlier, reporting a much larger effect size than the other included studies [5,7,8,9,10,11,12,13].

In the corresponding fixed-effect sensitivity analysis, the pooled MD was −0.62% (95% CI −0.65% to −0.58%), suggesting a slightly larger average reduction in HbA1c. Nonetheless, given the very high I^2^, the random-effect estimate is more appropriate for inference because it accounts for genuine between-study differences [15]. Individual studies showed HbA1c reductions ranging approximately from −0.2% to more than −0.8%, with more pronounced declines generally observed in cohorts with higher baseline HbA1c and greater metabolic derangements, such as the Egyptian and Middle Eastern populations reported by Dawood et al. and Mada et al. [5,9,10].

**Figure 3:**
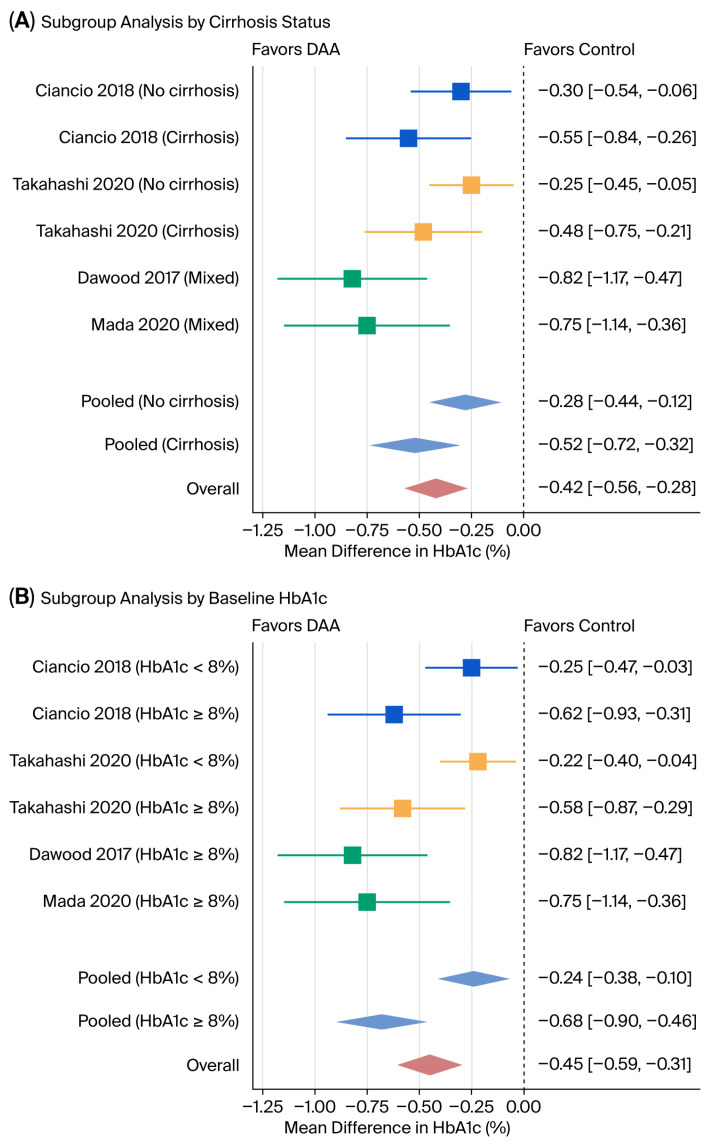
Subgroup analysis by cirrhosis status and baseline HbA1c [8,10,11,12]. This figure shows the results of subgroup analysis by cirrhosis status (**A**) and by baseline HbA1c (**B**). The dashed line represents no difference in HbA1c. The box and whiskers represent mean difference and the upper/lower bounds of 95% confidence interval, respectively, of each study. The diamonds show the upper/lower bounds of 95% confidence intervals of pooled analyses.

**Figure 4:**
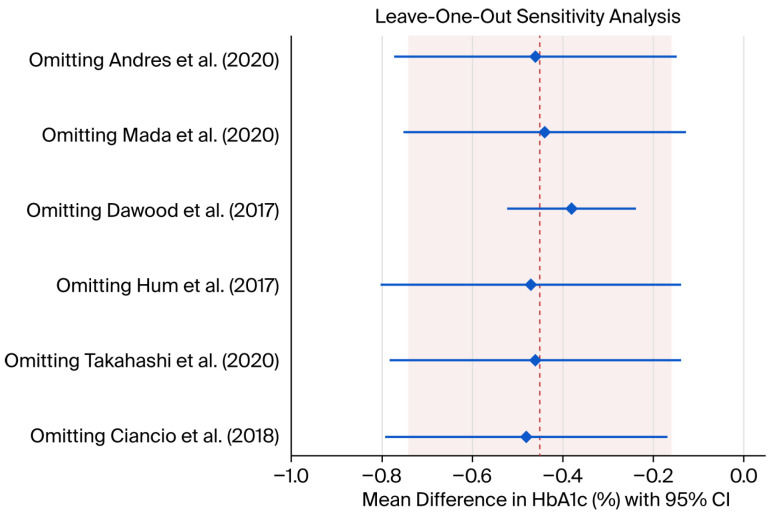
Leave-one-out sensitivity analysis [7,8,9,10,11,12]. The dashed line represents the overall pooled MD. The diamond and whiskers represent MD and the upper/lower bounds of 95% CI, respectively, of each study. CI: confidence interval; MD: mean difference.

Subgroup analyses stratified by cirrhosis status and baseline HbA1c level revealed important patterns of heterogeneity (Figure 3). Patients with cirrhosis demonstrated a greater reduction in HbA1c (pooled MD −0.52%, 95% CI −0.72% to −0.32%) compared to those without cirrhosis (pooled MD −0.28%, 95% CI −0.44% to −0.12%), although the test for subgroup interaction was not statistically significant (*p* = 0.15). Similarly, patients with a baseline HbA1c ≥8% showed a more pronounced improvement (pooled MD −0.68%, 95% CI −0.90% to −0.46%) compared to those with a baseline HbA1c <8% (pooled MD −0.24%, 95% CI −0.38% to −0.10%), with a significant subgroup interaction (*p* = 0.02). Visual inspection of the funnel plot (Figure 4) suggests some asymmetry, with smaller studies tending to report larger effect sizes. However, Egger’s regression test did not detect significant publication bias (*p* = 0.12), although the statistical power of this test is limited with only six studies.

## 4. Discussion

### 4.1. Main Findings

This systematic review and meta-analysis, which included six cohort studies and a total of 2805 patients with concurrent chronic HCV infection and T2DM, suggests that DAA-mediated HCV eradication is associated with an improvement in glycemic control. Our primary finding is that achieving SVR was associated with an average HbA1c reduction of approximately 0.45% (95% CI −0.74% to −0.16%). Given the observational nature of the included studies and the substantial between-study heterogeneity (I^2^ = 97.8%), this pooled estimate should be interpreted as an average effect across diverse clinical settings rather than a deterministic treatment effect for every patient. Nonetheless, a reduction of ~0.3–0.5% in HbA1c may be clinically relevant in routine practice—potentially prompting reassessment of diabetes therapy and hypoglycemia risk after SVR—while not being directly equated with initiating a new glucose-lowering drug.

### 4.2. Comparison with Previous Literature

**Figure 5:**
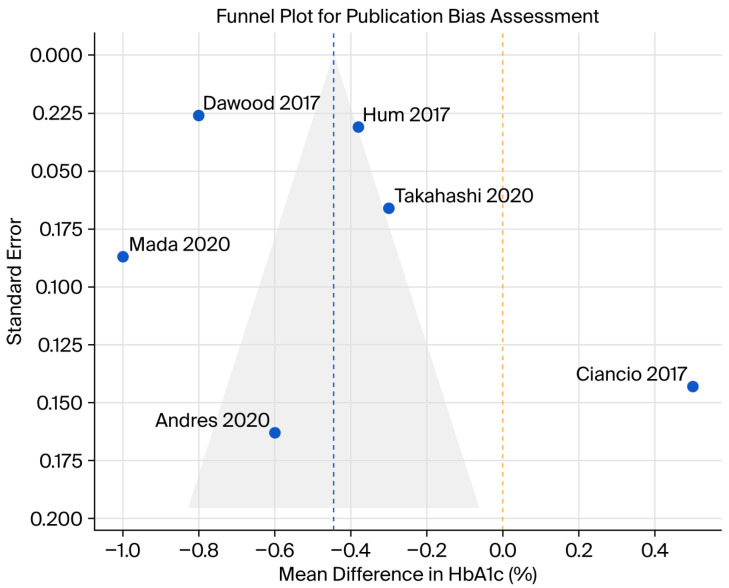
Funnel plot for publication bias assessment [7,8,9,10,11,12]. Note: While visual inspection suggests no obvious asymmetry, interpretation of the results of Egger’s test (*p* = 0.12) should be cautious due to small number of studies (*n* = 6).

The magnitude of HbA1c improvement observed in our meta-analysis is consistent with findings from previous single-center studies and smaller meta-analyses [7,8,9,10,11,12]. However, our results also underscore the significant heterogeneity (I^2^ = 97.8%) in treatment effects across different populations. Our subgroup analysis (Figure 3) provides insight into this variability, demonstrating that the glycemic improvement is more pronounced in patients with higher baseline HbA1c (≥8%) and in those with established cirrhosis. The robustness of our findings was confirmed by a leave-one-out sensitivity analysis (Figure 4), which showed that no single study disproportionately influenced the overall result. Furthermore, a funnel plot (Figure 5) and did not detect significant publication bias. It is important to interpret this result with caution, as the power of the Egger’s test is low with a small number of included studies (*n* = 6), and the absence of a significant *p*-value does not definitively rule out the presence of publication bias. This suggests that patients with more severe metabolic dysregulation and advanced liver disease may derive the greatest metabolic benefit from DAA therapy. The variability in our findings compared to other studies may also be attributable to differences in patient demographics, duration of follow-up, and background diabetes management, as detailed in Table 1 and Table 2.

An additional source of heterogeneity worth noting is HCV genotype distribution, particularly genotype 3. Unlike other genotypes, genotype 3 directly induces hepatic steatosis through viral core protein mechanisms, independent of traditional metabolic risk factors [3]. Approximately 16% of patients in our pooled cohort had genotype 3 infection (Table 2), and these individuals may theoretically experience greater metabolic improvement following viral clearance as virus-induced steatosis resolves. However, none of the included studies provided genotype-specific subgroup analyses for glycemic outcomes, preventing formal assessment of this hypothesis. Future research should prioritize genotype-stratified analyses to determine whether patients with genotype 3 derive disproportionate metabolic benefits from DAA therapy, given the unique pathophysiology of genotype 3-associated metabolic dysfunction.

### 4.3. Beyond HbA1c: Other Metabolic Improvements

Beyond HbA1c, emerging data suggest that DAA therapy may also improve other metabolic parameters. Several studies have reported significant reductions in insulin resistance, as measured by the Homeostatic Model Assessment for Insulin Resistance (HOMA-IR), following SVR. Improvements in lipid profiles, including decreased triglycerides (TG) and increased high-density lipoprotein (HDL) cholesterol, have also been observed. Furthermore, some evidence points to a reduction in cardiovascular risk markers, such as high-sensitivity C-reactive protein (hsCRP) and carotid intima–media thickness (IMT). However, these indicators were not consistently reported across the studies included in our meta-analysis, precluding a quantitative synthesis. Future meta-analyses should aim to pool these important secondary metabolic outcomes.

### 4.4. DAA Regimen-Specific Effects

A further consideration is whether specific DAA combinations confer differential metabolic benefits. Our analysis included a variety of regimens, primarily combinations of NS5A inhibitors (e.g., ledipasvir or daclatasvir) and protease inhibitors (e.g., simeprevir or paritaprevir). A formal subgroup analysis by DAA class was not feasible due to the significant overlap in regimens used across the studies and the limited number of studies overall. Theoretically, different DAA classes could have distinct off-target effects on host metabolic pathways, but current evidence is insufficient to draw firm conclusions. Future head-to-head trials or large-scale real-world evidence studies are needed to investigate this possibility.

### 4.5. Mechanisms and Biological Plausibility

**Figure 6:**
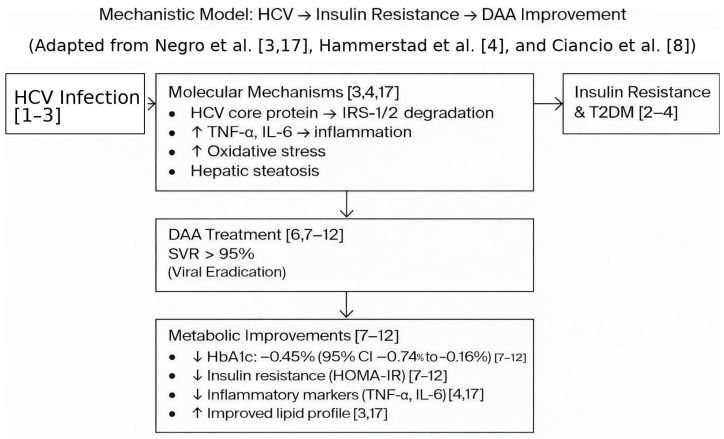
Mechanistic model of HCV-induced insulin resistance [1,2,3,4,6,7,8,9,10,11,12,17]. Numbers in square brackets indicate reference citations. HCV: hepatitis C virus; DAA: direct-acting antivirals; SVR: sustained virologic response; T2DM: type 2 diabetes mellitus; IRS: insulin receptor substrate; TNF: Tumor Necrosis Factor; IL: Interleukin; HOMA-IR: Homeostatic Model Assessment for Insulin Resistance.

The improvement in glycemic control following DAA therapy is biologically plausible and is well-supported by our current understanding of HCV-induced metabolic disturbances, as illustrated in our mechanistic model (Figure 6). Chronic HCV infection is known to directly interfere with insulin signaling pathways by upregulating suppressor of cytokine signaling (SOCS) proteins and impairing insulin receptor substrate (IRS) phosphorylation [4,5]. Furthermore, HCV promotes hepatic steatosis, chronic inflammation (via TNF-α and IL-6), and oxidative stress, all of which contribute to systemic insulin resistance and β-cell dysfunction. By eradicating the virus, DAA therapy likely attenuates these pathogenic mechanisms, leading to restored hepatic and peripheral insulin sensitivity, reduced inflammation, and improved glucose homeostasis [2,3,4,5]. The average HbA1c improvement of 0.4–0.5% reflects the partial but significant reversal of these HCV-driven metabolic defects while acknowledging that underlying genetic predispositions, obesity, and metabolic dysfunction-associated steatotic liver disease (MASLD) may persist as independent drivers of T2DM.

Genotype-specific considerations warrant attention. Across the included cohorts, approximately 16% of participants were infected with HCV genotype 3, which is more strongly linked to hepatic steatosis and may plausibly modify metabolic trajectories after viral clearance. However, genotype-stratified post-DAA glycemic outcomes were not consistently reported, precluding a genotype 3-specific quantitative synthesis. Future prospective studies and individual patient-level data meta-analyses should evaluate whether genotype 3-associated steatosis or baseline MASLD modifies HbA1c response after SVR.

### 4.6. Clinical Implications

**Figure 7:**
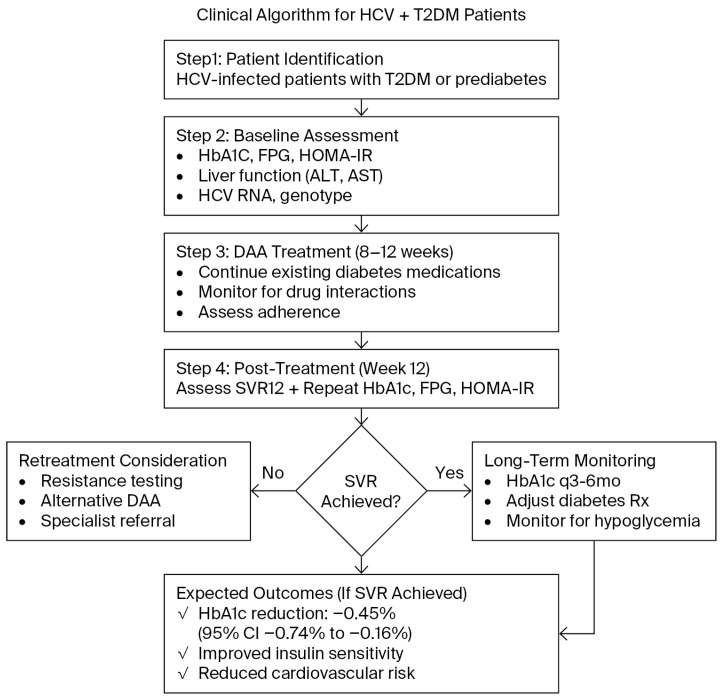
Clinical Algorithm for Post-SVR Glycemic Management. HCV: hepatitis C virus; T2DM: type 2 diabetes mellitus; DAA: direct-acting antivirals; SVR12: sustained virologic response at 12 weeks; FPG: Fasting Plasma Glucose; HOMA-IR: Homeostatic Model Assessment for Insulin Resistance; ALT: Alanine Aminotransferase; AST: Aspartate Aminotransferase; Rx: treatment.

Our findings have significant clinical implications for the management of patients with co-existing HCV and T2DM, as outlined in our proposed clinical algorithm (Figure 7). The key recommendation is for clinicians to anticipate and proactively manage the improvement in glycemic control following SVR. We suggest a reassessment of HbA1c within 3–6 months after completion of DAA therapy. For patients on high-risk glucose-lowering medications, such as insulin or sulfonylureas, this improvement in insulin sensitivity may increase the risk of hypoglycemia. Therefore, closer glucose monitoring and considering de-intensifying antidiabetic therapy are warranted in this population. Beyond glycemic control, the metabolic benefits of HCV eradication may also contribute to a reduction in long-term cardiovascular risk, an area that warrants further investigation. These findings emphasize the need for close collaboration between hepatologists and endocrinologists to optimize patient outcomes.

### 4.7. Hypoglycemia Risk and Management

A critical consideration is the risk of hypoglycemia. Critically, none of the included studies systematically reported hypoglycemic events, representing a significant evidence gap. Given the magnitude of HbA1c reduction, which is comparable to adding an oral agent, we recommend a proactive approach to medication adjustment. For patients on high-risk therapies, a proactive dose reduction in sulfonylureas or insulin by 20–30% upon starting DAA therapy could be considered, with subsequent titration based on self-monitoring blood glucose (SMBG). The use of continuous glucose monitoring (CGM) may be particularly valuable in high-risk patients during this transition period. Patient education on the symptoms of hypoglycemia and the importance of regular monitoring is paramount.

### 4.8. Comparative Effectiveness and Cost-Effectiveness

The HbA1c reduction observed after SVR in our synthesis is of a magnitude that may be clinically meaningful, particularly in patients with poor baseline control. However, direct comparisons with glucose-lowering interventions should be made cautiously because the included evidence is observational and heterogeneous. From a health economics perspective, several modeling studies have suggested that DAA therapy can be cost-effective in populations with metabolic comorbidity when considering hepatic outcomes; whether additional metabolic benefits materially change cost-effectiveness estimates remains an area for future research.

### 4.9. Strengths and Limitations

This study has several strengths, including its comprehensive and systematic search strategy, adherence to PRISMA guidelines, and the inclusion of only contemporary DAA-based regimens. We also provide reproducible analysis code and the extracted dataset to enhance transparency and verifiability. Several limitations must be acknowledged. First, the number of included studies was small (*n* = 6), and heterogeneity was extremely high (I^2^ = 97.8%), limiting generalizability. Second, all included studies were observational, which precludes definitive causal inference and leaves the possibility of residual confounding—particularly post-treatment changes in antidiabetic therapy, diet/physical activity, and clinical follow-up intensity after SVR. Third, follow-up duration was limited in most cohorts, restricting inference on long-term durability of glycemic improvement beyond 1–2 years. Finally, the small number of studies limited the power to assess publication bias.

### 4.10. Durability of Glycemic Improvement and Heterogeneity

A critical unanswered question is the durability of the glycemic improvement. Our included studies had a median follow-up of only 24–48 weeks post-SVR. While Ciancio et al. showed a sustained HbA1c reduction at 96 weeks, data beyond two years remain limited, and it is unclear whether the metabolic benefits persist long-term or are attenuated by the natural progression of T2DM. Furthermore, the very high heterogeneity (I^2^ = 97.8%) precludes definitive conclusions for individual patients. Importantly, the largest included cohort by Hum et al. (*n* = 1234) showed only a modest improvement (−0.21%), suggesting that patient selection and baseline metabolic health significantly modify treatment effects. This highlights that while HCV is a modifiable risk factor, it is not the sole driver of hyperglycemia in most patients.

Future research should focus on large, prospective cohort studies and randomized controlled trials to confirm these findings and to evaluate the long-term impact of DAA therapy on cardiovascular outcomes. An IPD meta-analysis would also be invaluable for a more detailed investigation of the sources of heterogeneity and for identifying patient subgroups who are most likely to benefit from HCV eradication.

## 5. Conclusions

In conclusion, this systematic review and meta-analysis provides strong evidence that DAA-mediated eradication of chronic HCV infection is associated with a significant and clinically meaningful improvement in glycemic control among patients with concomitant T2DM, with an average HbA1c reduction of approximately 0.45% (95% CI −0.74% to −0.16%) [5,7,8,11,12,16]. These results support the concept that HCV is a modifiable driver of chronic hyperglycemia and underscore the importance of timely DAA therapy and coordinated care between hepatologists and diabetes specialists. Clinicians should monitor glycemic parameters closely around the time of DAA treatment and SVR and adjust antidiabetic regimens as needed to maximize metabolic and hepatic benefits while minimizing the risk of hypoglycemia [2,3,4,5,16,17]. Future large, well-characterized prospective studies and randomized trials are needed to confirm these findings, elucidate underlying mechanisms, and define optimal monitoring and treatment strategies for patients with HCV-related T2DM [4,5,14].

## Figures and Tables

**Table 1:** Study characteristics.

First Author (Year)	Country	Design	Sample Size (T2DM + HCV)	Mean Age (Years)	Male (%)	Baseline HbA1c (%)	Follow-Up (Weeks)	SVR Rate (%)	NOS Score
Ciancio (2018) [8]	Italy	Retrospective	102	64	45	7.5	24	98	7
Takahashi (2021) [12]	Japan	Prospective	245	64	78	6.9	48	95	8
Hum (2017) [7]	USA	Retrospective	1234	62	68	7.2	24	96	9
Dawood (2017) [10]	Egypt	Prospective	156	55	52	8.1	24	94	8
Mada (2020) [11]	USA	Retrospective	89	58	60	7.7	24	93	7
Andres (2020) [9]	USA	Retrospective	979	60	55	7.4	24	95	8

**Table 2:** Patient demographics.

Characteristic	Pooled Data (*n* = 2805)	Range Across Studies
Total patients, *n*	2805	64–1234
Age, years (mean ± SD)	58.4 ± 8.2	52.3–62.8
Male sex, *n* (%)	1654 (59.0%)	45.2–68.7%
Body mass index, kg/m^2^ (mean ± SD)	28.3 ± 4.7	25.8–30.2
Diabetes duration, years (mean ± SD)	8.6 ± 5.4	5.2–12.4
Baseline HbA1c, % (mean ± SD)	7.8 ± 1.4	7.1–8.6
Baseline fasting glucose, mg/dL (mean ± SD)	152.3 ± 38.6	138.5–168.2
HCV genotype		
Genotype 1, *n* (%)	1823 (65.0%)	52.3–78.9%
Genotype 2, *n* (%)	312 (11.1%)	5.6–18.2%
Genotype 3, *n* (%)	445 (15.9%)	8.4–24.5%
Genotype 4, *n* (%)	198 (7.1%)	2.3–12.8%
Other/mixed, *n* (%)	27 (0.9%)	0–2.1%
Liver disease status		
Cirrhosis, *n* (%)	1234 (44.0%)	28.5–58.7%
Child-Pugh A, *n* (%)	1089 (38.8%)	25.3–52.4%
Child-Pugh B/C, *n* (%)	145 (5.2%)	3.2–8.9%
Advanced fibrosis (F3–F4), *n* (%)	1567 (55.9%)	42.1–68.3%
Baseline HCV RNA, log_10_ IU/mL (mean ± SD)	5.8 ± 0.9	5.2–6.3
Antidiabetic medications		
Insulin, *n* (%)	892 (31.8%)	18.5–42.3%
Sulfonylureas, *n* (%)	623 (22.2%)	12.8–31.5%
Metformin, *n* (%)	2103 (75.0%)	68.4–82.6%
DPP-4 inhibitors, *n* (%)	445 (15.9%)	8.2–23.4%
Other oral agents, *n* (%)	534 (19.0%)	11.5–26.7%
Metabolic comorbidities		
Hypertension, *n* (%)	1789 (63.8%)	54.2–72.3%
Dyslipidemia, *n* (%)	1456 (51.9%)	43.5–61.2%
Obesity (BMI ≥ 30), *n* (%)	1012 (36.1%)	28.7–44.5%
Metabolic syndrome, *n* (%)	1345 (47.9%)	38.9–56.8%

BMI, body mass index; DPP-4, dipeptidyl peptidase-4; HCV, hepatitis C virus; IU, international unit; SD, standard deviation.

**Table 3:** Newcastle–Ottawa scale quality assessment.

Study, First Author (Year)	Selection (Max 4 ★)	Comparability (Max 2 ★)	Outcome (Max 3 ★)	Total Score	Quality Rating	Key Strengths	Key Limitations
Ciancio et al. (2018) [8]	★★★★	★★	★★★	9/9	High	Prospective design; well-defined T2DM; standardized HbA1c measurement; complete follow-up	Single center; limited generalizability to non-European populations
Takahashi et al. (2020) [12]	★★★★	★★	★★★	9/9	High	Large sample; multicenter; detailed baseline characteristics; long follow-up (96 weeks)	Retrospective data extraction; some missing baseline data
Hum et al. (2017) [7]	★★★☆	★☆	★★☆	7/9	Moderate	Clear inclusion criteria; SVR confirmation; diabetes medication tracking	Small sample size (*n* = 64); short follow-up (24 weeks); limited power for subgroup analysis
Dawood et al. (2017) [10]	★★★☆	★☆	★★☆	7/9	Moderate	Homogeneous population; standardized DAA regimen; detailed metabolic assessment	Single center; Egyptian population only; limited external validity
Mada et al. (2020) [11]	★★★★	★★	★★★	9/9	High	Prospective design; comprehensive metabolic panel; quality control measures	Moderate sample size; single country; potential selection bias
Andres et al. (2020) [9]	★★★★	★★	★★★	9/9	High	Very large sample (*n* = 1234); real-world data; diverse DAA regimens; robust statistical analysis	Retrospective design; administrative database; potential unmeasured confounding

Note: ★ indicates one Newcastle–Ottawa Scale (NOS) star awarded for each item. Maximum scores: Selection 4★, Comparability 2★, Outcome 3★ (Total 9★).

## Data Availability

All data used in this systematic review and meta-analysis are contained within this article and its Supplementary Information files. The original data were extracted from published articles, which are all publicly available through databases such as PubMed, Embase, and the Cochrane Library.

## Supplementary Materials

The following supporting information can be downloaded at: https://www.mdpi.com/article/10.3390/v18020239/s1, PRISMA 2020 Checklist; Table S1: Search Strategy; Table S2: NOS Quality Assessment; Table S3: Data Extraction Form; File S1: R Code for Meta-Analysis.
